# Regulatory Roles of Circular RNAs in Coronary Artery Disease

**DOI:** 10.1016/j.omtn.2020.05.024

**Published:** 2020-05-23

**Authors:** Shuchen Zhang, Wenjing Wang, Xiaoguang Wu, Xiang Zhou

**Affiliations:** 1 Department of Cardiology, The Second Affiliated Hospital of Soochow University, Suzhou 215004, P.R. China

**Keywords:** circular RNAs, coronary artery disease, pathogenesis

## Abstract

Coronary artery disease (CAD) is a cardiac disorder caused by abnormal structure or function of the coronary artery, which leads to myocardial ischemia and hypoxia. CAD is a major cause of morbidity and mortality worldwide. Although there are currently effective drug therapies, there is a pressing need to find novel molecular therapeutic targets for CAD. The development of molecular biology technology has allowed the recognition of circular RNAs (circRNAs) as a novel class of noncoding RNAs that regulate gene function. The pathological roles of circRNAs in CAD have not, however, been comprehensively summarized. In this article, we review published research linking circRNAs to CAD and summarize the regulatory roles of circRNAs in the pathogenesis of coronary atherosclerosis, myocardial infarction, ischemia/reperfusion injury, and ischemic heart failure.

## Graphical Abstract

**Figure:**
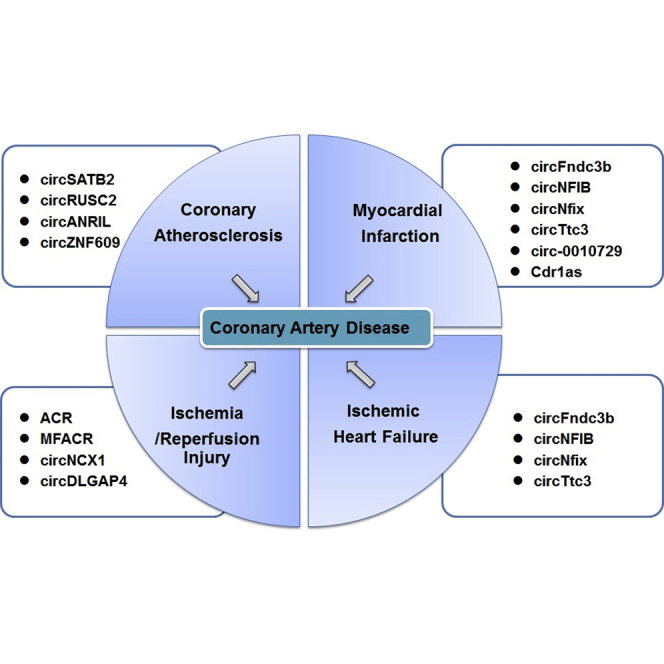

## Teaser

In this article, Zhou and colleagues review published research linking circRNAs to coronary artery disease and summarize the regulatory roles of circRNAs in the pathogenesis of coronary atherosclerosis, myocardial infarction, ischemia/reperfusion injury, and ischemic heart failure.

## Introduction

The incidence of coronary artery disease (CAD) is increasing rapidly and is adversely affecting people’s health and life expectancy. In CAD, the heart experiences decreased blood perfusion, with hypoxia or abnormal lipid metabolism, resulting in pathophysiological processes, such as apoptosis, autophagy, and fibrosis. Acute or chronic myocardial ischemia eventually leads to varying degrees of CAD, such as angina pectoris, myocardial infarction, and ischemic heart failure.^1^, ^2^, ^3^, ^4^

The protein-coding genes account for only 1%–2% of the human genome, whereas the majority of transcripts are noncoding RNAs (ncRNAs).^5^ Circular RNAs (circRNAs), a type of ncRNA, are conserved, covalently closed, single-stranded transcripts, with lengths ranging from dozens to thousands of base pairs, in which the 5′ and 3′ termini are covalently linked by backsplicing of exons from a single mRNA precursor.^6^ circRNAs, which play important roles in epigenetics and various cellular processes, exist dynamically and specifically in different cells and tissues, as well as in different species and at different stages of development.^7^ circRNAs can be formed by either lariat-driven circularization or intron pairing-driven circularization.^8^ After a series of reactions, including formation of lariat intermediates, internal splicing reactions, and debranching of the lariat, three different types of circular transcript can be formed: exon-only circRNAs (e-circRNAs; most often referred to as circRNAs), exon-intron circRNAs (e/i-circRNAs),^9^ and intron-only circRNAs (i-circRNAs).^10^ Different types of circRNA perform their physiological functions in different ways. For example, e-circRNAs can act as microRNA (miRNA, or miR) sponges,^6^^,^^11^^,^^12^ protein sponges,^13^^,^^14^ protein decoys,^15^ and scaffolds for the formation of protein complexes.^16^^,^^17^ Although most circRNAs are noncoding, some can be translated into proteins.^18^ Numerous e-circRNAs carry out their physiological roles by acting as miRNA and protein sponges, largely in the cytoplasm.^19^ Most i-circRNAs and e/i-circRNAs are reported to be concentrated in the nucleus and to play an indispensable role in regulating expression of their parent genes.^10^ Altesha et al.^20^ and Bei et al.,^21^ respectively, have described in detail the biogenesis and biological function of circRNAs.

**Figure 1:**
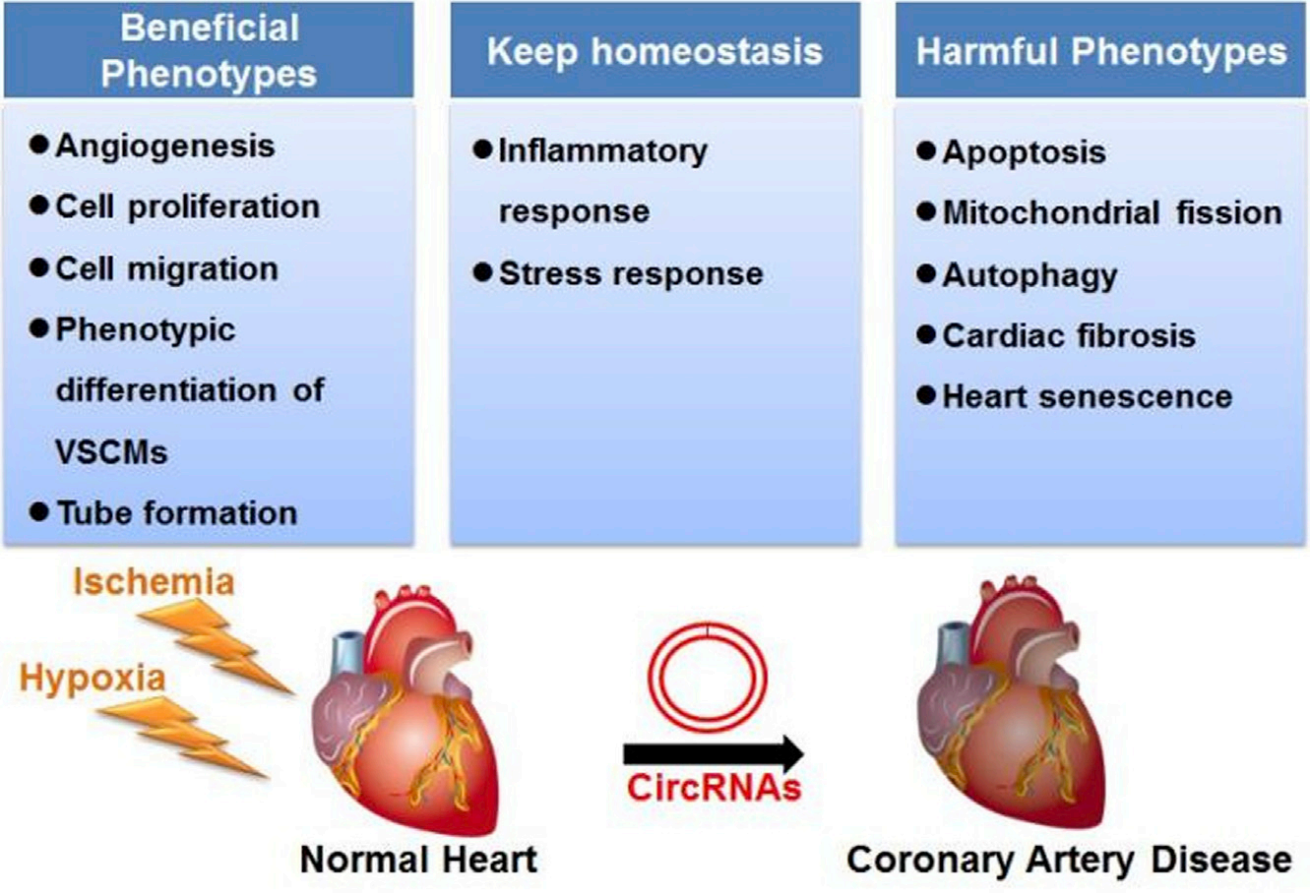
Involvement of circRNAs in Coronary Artery Disease (CAD) Some circRNAs improve the pathological outcome of ischemia by promoting angiogenesis, cell proliferation, and migration. Other circRNAs, however, accelerate apoptosis, autophagy, and myocardial fibrosis, thus worsening the outcome of CAD. Some circRNAs are responsible for maintaining homeostasis in CAD by regulating inflammatory and stress responses.

**Figure 2:**
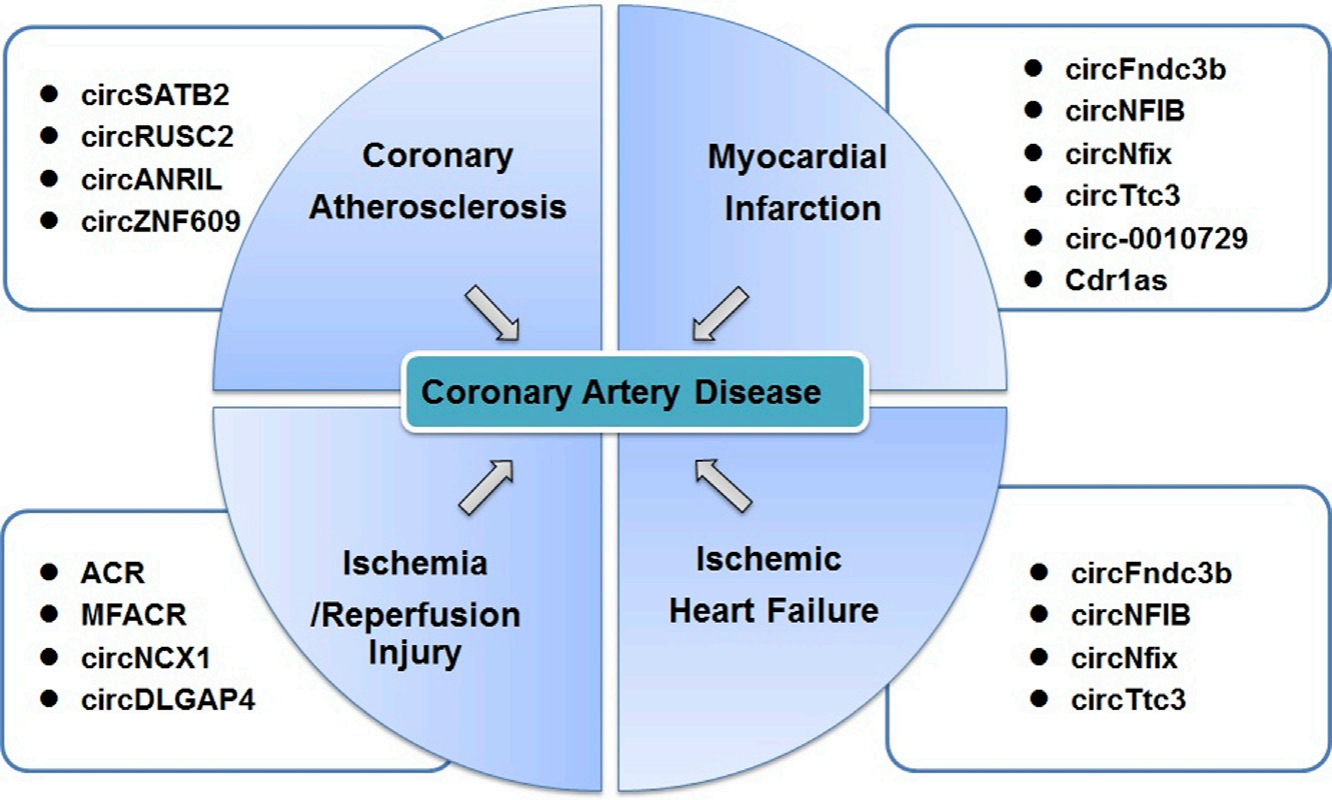
Regulatory Roles of circRNAs in CAD circRNAs are involved in the regulation of CAD, including coronary atherosclerosis, myocardial infarction, ischemia/reperfusion injury, and ischemic heart failure.

The incidence of CAD is increasing rapidly, and CAD has become the leading cause of morbidity and mortality worldwide. In recent years, accumulating evidence has demonstrated that circRNAs are critically involved in the pathogenesis of CAD (Figure 1). In this article, we review published research linking circRNAs to CAD and summarize the regulatory roles of circRNAs in promoting or inhibiting coronary atherosclerosis, myocardial infarction, ischemia/reperfusion injury, and ischemic heart failure (Figure 2).

### circRNAs and Coronary Atherosclerosis

Coronary atherosclerosis, which is caused by stenosis of the coronary artery by atherosclerotic lesions, results in myocardial ischemia, hypoxia, or necrosis. The transition between contractile vascular smooth muscle cells (VSMCs) and proliferative VSMCs that occurs during the progression of atherosclerosis may potentially provide a new molecular target for the treatment of CAD.^22^ Structural and functional changes in VSMCs are the cytopathological basis of CAD, and effective control of phenotypic changes in VSMCs can prevent CAD. Two studies have investigated the molecular mechanisms linking circRNAs to CAD. In the first study, differential expression of circSATB2 was observed in different categories of VSMCs in CAD, and circSATB2 was shown to exert the cardioprotective role by binding to miR-939, which has known negative effects on the pathogenesis of CAD.^23^ In a more recent study, circRUSC2 was found to enhance proliferation and migration of coronary VSMCs and to protect the cells from apoptosis by binding to miR-661 and thus, increasing expression of spleen tyrosine kinase (SYK).^24^

Different forms of transcript associated with a certain circRNA usually perform different functions. However, antisense noncoding RNA in the *INK4* locus (ANRIL), long noncoding ANRIL, and circANRIL are all closely related to cardiovascular disease. Expression of ANRIL has been confirmed in vascular endothelial cells and VSMCs,^25^ and a genetic association between long noncoding ANRIL and CAD has also been confirmed.^26^^,^^27^ In a rat model of coronary atherosclerosis, increased expression of circANRIL was shown to increase serum lipid levels and atherogenic index, as well as to enhance levels of inflammatory factors and promote apoptosis of vascular endothelial cells.^28^

hsa-circ-0001445, also referred to as cSMARCA5, is a circRNA derived from exons 15 and 16 of the SMARCA5 gene. Differential expression of cSMARCA5 has been found in hepatocellular carcinoma, cirrhosis, and hepatitis B patients.^29^^,^^30^ Plasma levels of hsa-circ-0001445 are lower in patients with higher coronary atherosclerotic burden. hsa-circ-0001445 is expressed in human coronary smooth muscle cells, and its secretion is reduced in atherosclerotic conditions.^31^ hsa-circ-0001879 and hsa-circ-0004104 were shown to be significantly upregulated in CAD patients, which sheds new light on the potential of circRNAs as biomarkers for diagnosing CAD.^32^ Microarray analysis of circRNAs showed that hsa-circ-11783-2 in peripheral blood is closely associated with both CAD and type 2 diabetes mellitus.^33^ hsa-circ-0124644 in the peripheral blood has also been identified as a diagnostic biomarker of CAD.^34^ Microarray analysis of competing endogenous RNA (ceRNA) revealed that nine circRNAs, including hsa-circ-0089378, hsa-circ-0083357, hsa-circ-0082824, hsa-circ-0068942, hsa-circ-0057576, hsa-circ-0054537, hsa-circ-0051172, hsa-circ-0032970, and hsa-circ-0006323, play an important role in promoting TRPM3 expression by inhibiting hsa-miR-130a-3p in CAD patients. TRPM3 acts as a target gene for hsa-miR-130a-3p and regulates cellular calcium homeostasis.^35^

In other vascular diseases, such as retinopathy of prematurity and diabetic retinopathy, silencing circZNF609 attenuated hypoxic and oxidative stress in endothelial cells, reduced retinal vessel loss, and inhibited pathological angiogenesis. circZNF609 is downregulated in blood samples of CAD patients,^36^ providing preliminarily confirmation of the feasibility of treating vascular diseases by altering expression of circRNA.

### circRNAs and Myocardial Infarction

Myocardial infarction is caused by continuous ischemia and hypoxia of the coronary artery, which can be accompanied by arrhythmia, shock, or heart failure and is often life threatening. Recently, growing evidence has indicated that circRNAs can participate in regulating the pathophysiological process of myocardial infarction.

In a mouse model of myocardial infarction, circFndc3b, transcribed from its parent gene Fndc3b, was significantly downregulated in heart tissues 3 days post-myocardial infarction compared with the sham group. Mechanistically, circFndc3b was shown to act as an endogenous sponge of the RNA binding protein and to regulate the expression of proteins associated with the functions of cardiomyocytes, cardiac vascular endothelial cells, and fibroblasts following myocardial infarction. Ultimately, upregulation of circFndc3b may reduce infarct size and enhance angiogenesis.^37^

Similar to the transition of VSMCs in coronary atherosclerosis, there is a transformation of myofibroblasts during the progression of heart failure caused by myocardial infarction. Cardiac fibrosis is critically involved in pathological remodeling,^38^ and two types of fibrosis, termed reparative fibrosis and reactive fibrosis, coexist during myocardial remodeling.^39^ Local scar formation at the site of fibrosis, which is activated by a series of changes in α-smooth muscle actin and extracellular collagen during the development of myocardial infarction, can promote the repair of myocardial injury and prevent heart rupture.^40^ Cardiac fibrosis, however, eventually damages the structure of the heart, leading to systolic and diastolic dysfunction.^41^^,^^42^ Overexpression of circNFIB, which represses miR-433, attenuates proliferation of cardiac fibroblasts and differentiation of myofibroblasts and may counterbalance cardiac fibrosis.^43^ Therefore, circRNAs could be novel molecular targets for ventricular remodeling due to myocardial fibrosis.

**Figure 3:**
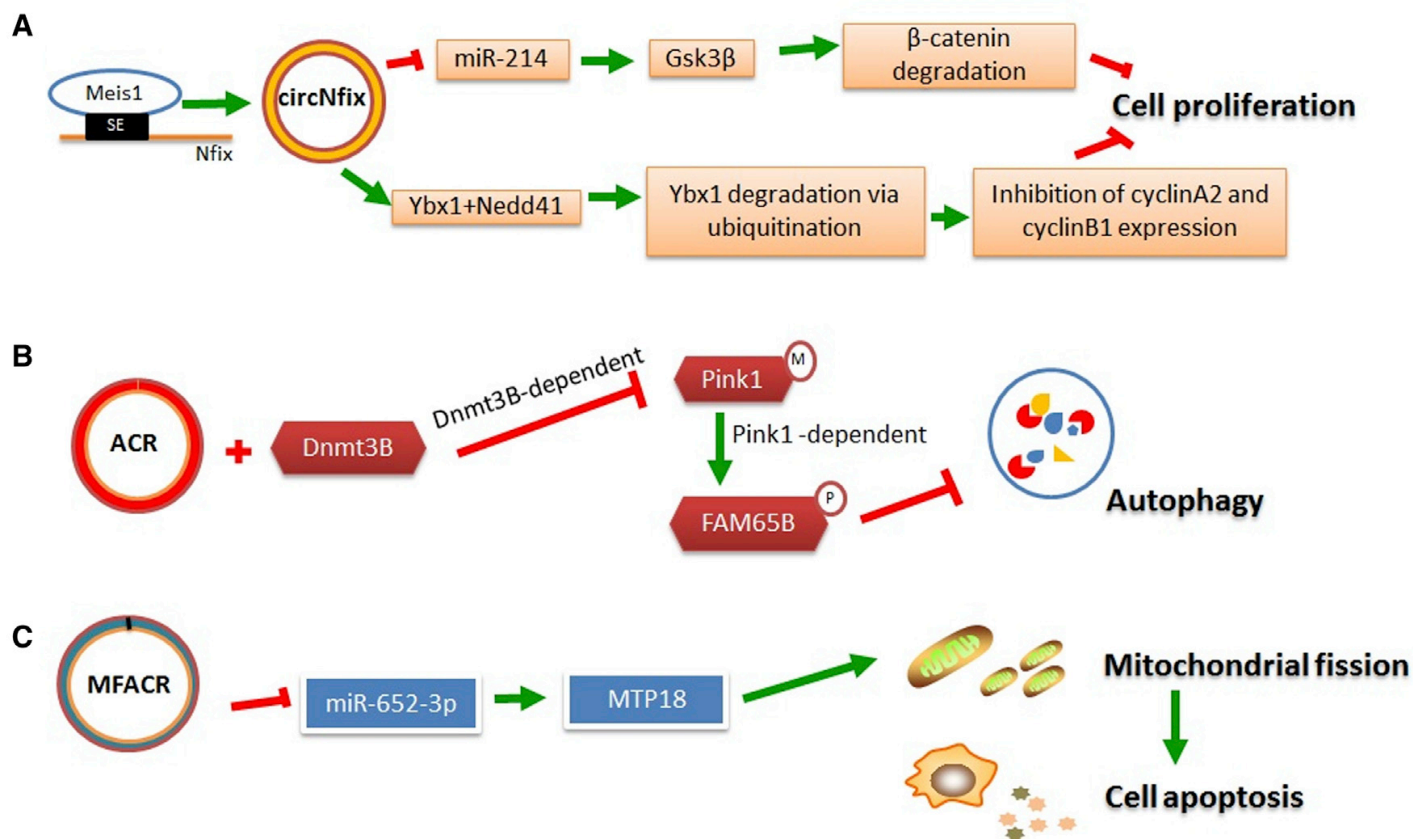
Schematic Representation of Mechanisms of Selected circRNAs in CAD (A) circNfix leads to the degradation of β-catenin and inhibits cell proliferation through combining with miR-214. Formation of the circNfix/Ybx1/Nedd4l complex promotes Ybx1 degradation through the ubiquitination-proteasome pathway. (B) The combination of ACR and Dnmt3B blocks Dnmt3B-dependent methylation of Pink1, resulting in enhanced phosphorylation of FAM65B and reduced autophagy. (C) By acting as a miR-652-3p sponge, MFACR increases mitochondrial fission and cardiomyocyte apoptosis by elevating expression of MTP18, which is a downstream target of miR-652-3p.

circNfix, also known as mmu-circ-0001704, which acts as an endogenous sponge of miRNAs, needs additional regulation by the super-enhancer of the Nfix gene. Mechanistically, circNfix-associated super-enhancer regulates circNfix expression by recruiting Meis1, a key transcription factor controlling cardiomyocyte cell-cycle arrest, and Ybx1 becomes a substrate for the E3 ubiquitin ligase Nedd4l in the presence of circNfix.^44^ The formation of the circNfix/Ybx1/Nedd4l ternary complex arrests Ybx1 in the cytoplasm and promotes Ybx1 degradation through ubiquitination-proteasome pathways (Figure 3A). Cardiomyocyte-specific knockdown of circNfix increases expression of Ybx1 and miR-214 and leads to reduced apoptosis and infarct size and enhanced cardiomyocyte proliferation and cardiac regeneration.

circTtc3 is expressed at the same level in both cardiomyocytes and cardiac fibroblasts and is significantly upregulated in a rat model of myocardial infarction. miR-15 is sequestered with circTtc3, which reduces its regulatory effect on the Arl2 gene. Arl2 protein plays a key role in oxidative phosphorylation and ATP production.^45^ Collectively, the interaction of circTtc3, miR-15b, and Arl2 has a protective effect on the infarcted heart.^46^ circ-0010729, which negatively regulates miR-145-5p, is significantly overexpressed in human cardiomyocytes suffering from oxygen-glucose-deprivation injury. Overexpression of circ-0010729 suppresses cell viability and migration, resulting in cardiomyocyte apoptosis. As well as playing a role in several development processes involved in cell growth,^47^^,^^48^ the mammalian target of rapamycin (mTOR) signaling pathway has been shown to be cardioprotective following myocardial injury.^49^^,^^50^ The mitogen-activated protein kinase kinase/extracellular signal-regulated kinase (MEK/ERK) pathway has a similar protective effect following myocardial ischemic injury.^51^^,^^52^ The silencing of circ-0010729 has been found to prevent apoptosis and promote cell growth and migration by increasing expression of miR-145-5p and thus, enhancing the activity of the mTOR and MEK/ERK pathways.^53^ Both SP1, which is a transcription factor implicated in hypoxic gene transcription,^54^ and poly ADP ribose polymerase (PARP), which has a role in apoptosis induced by ischemia/reperfusion,^55^ are target genes of miR-7. Overexpression of Cdr1as, also known as ciRS-7, which is a sponge for miR-7, increases expression of SP1 and PARP, which jointly promote cardiomyocyte apoptosis during the progression of myocardial infarction.^56^

### circRNAs and Ischemia/Reperfusion Injury

Ischemia/reperfusion injury refers to the progressive aggravation of tissue injury caused by vascular recanalization after a period of partial or complete acute coronary artery occlusion, although the ischemic myocardium may be returned to normal perfusion. A series of traumatic changes in myocardial ultrastructure, energy metabolism, cardiac function, and electrophysiology is more prominent after vascular recanalization, and serious arrhythmias can even lead to sudden death.

It has recently been discovered that a novel circRNA, autophagy-related circRNA (ACR), has a cardioprotective function.^57^ The combination of ACR and Dnmt3B, which is a member of the DNA methyltransferase family, blocks Dnmt3B-dependent DNA methylation of the Pink1 promoter. ACR thus prevents degradation of the Pink1 promoter and subsequently contributes to Pink1-dependent methylation of FAM65B.^57^ Pink1, which localizes in the cytoplasm, functions as a mitochondria-related kinase^58^ and maintains the normal morphology of mitochondria.^59^ More importantly, Pink1 regulates the function of its downstream elements via phosphorylation.^60^ FAM65B is related to the small G protein RhoA^61^ and is reported to play an important role in many cellular processes, including cell adhesion, polarization, migration, and differentiation.^62^, ^63^, ^64^ The alteration of autophagy activity may exert cardioprotective effects during ischemia/reperfusion,^65^ heart failure,^66^ and ventricular remodeling.^67^ This study confirmed that the ACR-Pink1-FAM65B cascade can inhibit autophagy and shed new light on a potential molecular mechanism for the treatment of ischemia/reperfusion injury (Figure 3B).

Knockdown of mitochondrial fission and apoptosis-related circRNA (MFACR), also known as mm9-circ-016597, increases the expression of miR-652-3p and thus, inhibits the function of MTP18 (mitochondrial protein, 18 kDa), which is encoded by the nuclear gene MTP18.^68^ The mitochondrial membrane protein MTP18 plays a role in mitochondrial fission in many cell types.^69^^,^^70^ MFACR has 15 binding sites that are complementary to miR-652-3p and can repress the beneficial effects of miR-652-3p by acting as an endogenous sponge. In this study, Wang et al.^68^ confirmed that miR-652-3p inhibits the function of MTP18 by binding to its mRNA. This reaction cascade has a role in attenuating mitochondrial fission and apoptosis and protects cardiomyocytes and the heart from hypoxia and ischemia/reperfusion injury (Figure 3C).

circNCX1, which is transcribed from the ncx1 gene, was found to be significantly increased during oxidative stress, caused either by treating neonatal rat cardiomyocytes with H_2_O_2_ or by subjecting mice to myocardial ischemia/reperfusion. circNCX1 represses the activity of miR-133a-3p, which attenuates the negative effect of CDIP1, a nuclear gene that promotes apoptosis. The circNCX1-miR-133-3p-CDIP1 axis might exert a considerable effect on oxidative-induced apoptosis of cardiomyocytes.^71^ In addition, a recent study by Wang et al.^72^ suggested that circRNA DLGAP4 could attenuate cardiomyocyte apoptosis through regulating BCL2 via targeting miR-143 in myocardial ischemia-reperfusion injury.

### circRNAs and Ischemic Heart Failure

Ischemic heart failure, brought about by long-term myocardial ischemia, due to coronary atherosclerosis, is a common complication of CAD. Myocardial fibrosis and cardiac remodeling are important mechanisms for the development of this syndrome. With the increasing incidence of CAD, ischemic heart failure poses a growing threat to human health.

Garikipati et al.^37^ showed that the human homolog of circFndc3b was significantly downregulated in samples of cardiac tissue from ischemic cardiomyopathy patients. In the mouse model of myocardial infarction, overexpression of circFndc3b was found to reduce cardiomyocyte apoptosis, enhance angiogenesis, and improve cardiac function.^37^ Cardiac fibrosis is involved in adverse ventricular remodeling and is associated with mortality in patients with heart failure.^73^^,^^74^ Forced expression of circNFIB counterbalances cardiac fibrosis in heart failure after myocardial infarction by sponging miR-433.^43^ circNfix has been shown to inhibit the proliferation of cardiomyocytes by promoting degradation of Ybx1 via the ubiquitination-proteasome pathways, demonstrating that circNfix is a new therapeutic target for ischemic heart failure.^44^ circTtc3, acting as an endogenous sponge for miR-15b-5p, attenuates cardiomyocyte apoptosis and mitigates the pathologic progression of ischemia-associated heart failure.^46^

### Conclusions and Future Directions

In this review, we mainly summarized the regulatory roles of circRNAs in the pathogenesis of CAD (Table 1). From the studies looking at coronary atherosclerosis, we can surmise that circRNAs can be used as diagnostic biomarkers for CAD. Some circRNAs also shed light on disease progression at a molecular level, since their overexpression or knockout can significantly allay the pathological process, providing forward-looking guidance for the treatment of CAD. For example, circSATB2 and circRUSC2 can inhibit the phenotypic differentiation of VSMCs and apoptosis and promote cell proliferation and migration in the development of atherosclerosis. Downregulation of circANRIL attenuates cell apoptosis and maintains the balance of normal inflammatory response. From the studies on myocardial infarction, we learn that circRNAs, such as circFndc3b, circTtc3, circ-0010729, and Cdr1as, can regulate the apoptosis of cardiomyocytes. During ischemia/reperfusion injury, ACR, MFACR, and circNCX1 are involved in the regulation of cardiomyocyte autophagy and apoptosis. Based on the above research results, we believe that circRNAs will become emerging molecular targets for the treatment of CAD.

**Table 1:** Summary of Circular RNAs Associated with Coronary Artery Disease

Coronary Artery Disease	Circular RNAs	Host Gene	Type of Experiment	Differential Expression	Major Function	References
Coronary atherosclerosis	circSATB2 (hsa-circ-0007422)	SATB2	*vitro*	upregulated in proliferative VSMCs	regulate VSMC phenotypic differentiation, proliferation, migration, and apoptosis	^23^
	circRUSC2	RUSC2	*vitro*	upregulated in proliferative VSMCs	regulate VSMC phenotypic differentiation, proliferation, migration, and apoptosis	^24^
	circANRIL	ANRIL	*vivo*	upregulated	induce apoptosis and involve inflammatory response	^28^
	hsa-circ-0001445	SMARCA5	blood sample	downregulated	diagnostic biomarker	^31^
	hsa-circ-0001879 and hsa-circ-0004104	–	blood sample	upregulated	diagnostic biomarker	^32^
	hsa-circ-11783-2	–	blood sample	downregulated	diagnostic biomarker	^33^
	hsa-circ-0124644	–	blood sample	upregulated	diagnostic biomarker	^34^
	hsa-circ-0089378, etc.	–	blood sample	upregulated	regulate hsa-miR-130a-3p and TRPM3	^35^
	circZNF609	ZNF609	*vitro*/*vivo*	downregulated	involve the pathogenesis of vascular dysfunction	^36^
Myocardial infarction	circFndc3b	Fndc3b	*vitro*/*vivo*	downregulated	reduce cardiomyocyte apoptosis and enhance neovascularization	^37^
	circNFIB	NFIB	*vitro*/*vivo*	downregulated	attenuate cardiac fibrosis	^43^
	circNfix (mmu-circ-0001704)	Nfix	*vitro*/*vivo*	upregulated	inhibit cardiomyocyte proliferation and angiogenesis and promote cardiac dysfunction	^44^
	circTtc3	TTC3	*vitro*/*vivo*	upregulated	protect cardiomyocyte from apoptosis and ATP shortage	^46^
	circ-0010729	HSPG2	*vitro*	upregulated	aggrandize oxygen glucose deprivation-induced cell injury	^53^
	Cdr1as (ciRS-7)	CDR1 protein-coding gene	*vitro*/*vivo*	upregulated	promote cell apoptosis and increase cardiac infarct size	^56^
Ischemia/reperfusion injury	ACR (mmu-circ-006636)	–	*vitro*/*vivo*	downregulated	suppress autophagy	^57^
	MFACR (mm9-circ-016597)	Smyd4	*vitro*/*vivo*	upregulated	promote mitochondrial fission and apoptosis	^68^
	circNCX1	ncx1	*vitro*/*vivo*	upregulated	promote cell apoptosis	^71^
	circDLGAP4	–	*vivo*	downregulated	ameliorate cardiomyocyte apoptosis	^72^

VSMC, vascular smooth muscle cell.

In gathering the data for this review, we also identified several characteristics of circRNAs. First, increased expression of circRNAs under pathological conditions is mostly harmful. Second, most circRNAs are differentially expressed under pathological conditions, whereas the linear transcripts and the parent genes are not. Third, most circRNAs act as competing endogenous RNAs, whereas some others can function as protein sponges or participate in protein complexes. Fourthly, some circRNAs and their target miRNAs reciprocally regulate expression, whereas others do not. Finally, circRNAs can regulate the function of their targets by controlling phosphorylation, ubiquitination, and methylation.

Although research on circRNA is relatively mature, and there have been many publications, details of amplification, such as standardization of primer design, have not been formally explained in published articles because of the unusual splicing mode of circRNAs. Some studies have concluded that circulating circRNAs can be used as biomarkers for the diagnosis of CAD, but the sample size of these studies is relatively small. In addition, we need to explain that some circRNAs associated with intervention therapy were mainly derived from animal experiments, so the safety and applicability of viruses used to deliver circRNAs to humans must be taken into account. In the future, we will be able to further classify circRNAs acting on the cardiovascular system and explore whether there are organ-specific-expressed circRNAs, as judged by their effects on cardiomyocytes, endothelial cells, and VSMCs. A better understanding of these issues will enable precise treatment of cardiovascular diseases.

In summary, we have reviewed the involvement of circRNAs in the pathogenesis of CAD, which may provide valuable insights into the occurrence and development of CAD. We can foresee that circRNAs will become potential molecular targets for the treatment of CAD.

## Author Contributions

S.Z. and W.W. summarized the data and wrote the manuscript. X.W. performed the literature search. X.Z. revised the manuscript.

## Conflicts of Interest

The authors declare no competing interests.
